# Profile Hidden Markov Models for the Detection of Viruses within Metagenomic Sequence Data

**DOI:** 10.1371/journal.pone.0105067

**Published:** 2014-08-20

**Authors:** Peter Skewes-Cox, Thomas J. Sharpton, Katherine S. Pollard, Joseph L. DeRisi

**Affiliations:** 1 Biological and Medical Informatics Graduate Program, University of California San Francisco, San Francisco, California, United States of America; 2 Departments of Medicine, Biochemistry and Biophysics, and Microbiology, University of California San Francisco, San Francisco, California, United States of America; 3 The J. David Gladstone Institutes, University of California San Francisco, San Francisco, California, United States of America; 4 Institute for Human Genetics & Division of Biostatistics, University of California San Francisco, San Francisco, California, United States of America; 5 Howard Hughes Medical Institute, Bethesda, Maryland, United States of America; The University of Hong Kong, Hong Kong

## Abstract

Rapid, sensitive, and specific virus detection is an important component of clinical diagnostics. Massively parallel sequencing enables new diagnostic opportunities that complement traditional serological and PCR based techniques. While massively parallel sequencing promises the benefits of being more comprehensive and less biased than traditional approaches, it presents new analytical challenges, especially with respect to detection of pathogen sequences in metagenomic contexts. To a first approximation, the initial detection of viruses can be achieved simply through alignment of sequence reads or assembled contigs to a reference database of pathogen genomes with tools such as BLAST. However, recognition of highly divergent viral sequences is problematic, and may be further complicated by the inherently high mutation rates of some viral types, especially RNA viruses. In these cases, increased sensitivity may be achieved by leveraging position-specific information during the alignment process. Here, we constructed HMMER3-compatible profile hidden Markov models (profile HMMs) from all the virally annotated proteins in RefSeq in an automated fashion using a custom-built bioinformatic pipeline. We then tested the ability of these viral profile HMMs (“vFams”) to accurately classify sequences as viral or non-viral. Cross-validation experiments with full-length gene sequences showed that the vFams were able to recall 91% of left-out viral test sequences without erroneously classifying any non-viral sequences into viral protein clusters. Thorough reanalysis of previously published metagenomic datasets with a set of the best-performing vFams showed that they were more sensitive than BLAST for detecting sequences originating from more distant relatives of known viruses. To facilitate the use of the vFams for rapid detection of remote viral homologs in metagenomic data, we provide two sets of vFams, comprising more than 4,000 vFams each, in the HMMER3 format. We also provide the software necessary to build custom profile HMMs or update the vFams as more viruses are discovered (http://derisilab.ucsf.edu/software/vFam).

## Introduction

Viral infections are a major global health concern, and new infectious diseases continue to emerge [1], [2]. Emerging infectious diseases are a tremendous burden on economies and public health, and because many cases arise with no known etiology, there is a high demand for advances in viral diagnostic methods [2], [3]. Detection of viruses in clinical specimens traditionally depends on amplification of conserved regions of nucleic acid from viral genomes, immunological detection, or *in vitro* replication of virus in cell culture [1]. Though these traditional tests are highly specific and have been used for decades, they have major limitations. In particular, detection of novel, divergent, elusive, or low copy number viral genes within a complex host genetic background can be quite difficult using traditional tools such as PCR, conventional sequencing technologies, and even DNA microarrays [4], [5]. These limitations can be overcome by deep sequencing primary human samples, such as tissue, cerebral spinal fluid, bronchial or nasal lavage, and stool. Samples from non-sterile locations may contain nucleic acid from numerous commensal organisms, and the direct sequencing of all nucleic acid species of a specimen can elucidate the specimen’s metagenome, *i.e.*, the sequences derived from all the organisms present in the specimen [6], [7]. Thus, shotgun metagenomic sequencing for viral discovery necessitates identification of specific RNA or DNA sequences in the context of a complex and potentially unknown background of irrelevant nucleic acid.

Due to the decreasing cost and increasing throughput of second-generation sequencing technologies, deep sequencing of metagenomes and metatranscriptomes has become a critical tool for the identification of novel or divergent viruses that are difficult to detect by other methods [8], [9]. For example, massively parallel sequencing has been used to discover a variety of novel viruses, from a novel member of the *Bornaviridae* family in birds, to novel members of the *Arenaviridae* family in snakes [10]–[15]. Massively parallel sequencing can help overcome the problem of detecting pathogens present at vanishingly low amounts; traditional Sanger sequencing approaches will not provide ample sequencing depth to detect the pathogen because the proportion of metagenomic data deriving from a target pathogen may be on the order of one in one hundred thousand reads or lower [10]. A single deep sequencing experiment can now generate billions of sequencing reads, each of which is hundreds of nucleotides long. As generating viral sequence from metagenomes becomes more commonplace, there still remains the bioinformatic challenge of actually identifying those sequences, especially when the virus present is only distantly related to known viruses.

Viruses are typically identified in metagenomic sequencing datasets via homologous inference from sequence alignment. Common tools for this purpose include BLAST [16], BLAT [17], Bowtie [18], [19], or other pairwise sequence aligners. BLAST is quick and specific, and can find homologous protein pairs when the percent sequence identity of the alignment exceeds 30% over the length of a protein or protein domain. But as the pairwise sequence identity drops below 30% for full-length protein sequences, BLAST finds fewer true homologs [20], [21], and this problem is further compounded by the shorter reads generated by second-generation sequencers. Because pairwise alignment is limited in detecting low percent identity homologs, researchers seeking more distant evolutionary relationships have shifted towards so-called “profile” methods for the detection of remote homologs. Profile methods consider information across a family of evolutionarily related sequences, derived from a multiple sequence alignment. Profile search methods gain sensitivity by incorporating position-specific information into the alignment process and by quantifying variation across family members at each position [22]–[29]. For example, a query sequence can match a family profile because it is evolving like other members of the family, even if it is not significantly similar to any one known member of the family. Of the profile methods, profile hidden Markov models (profile HMMs) typically outperform other profile methods (*e.g.*, PSI-BLAST) in the detection of distant homologs [21]. Because many viruses have more error-prone polymerases than typically found in cellular organisms, especially RNA viruses that rely on RNA-dependent RNA-Polymerases (RdRP) for genome replication, more distant viral homologs can arise on much shorter evolutionary time-scales than are generally observed in bacteria or macroorganisms. In light of this higher mutation rate, profile HMMs are particularly well suited for the detection of divergent viral sequences.

Profile HMMs have been built for some viral proteins, but viral genes are not comprehensively covered in publicly available profile HMM databases. For example, we estimate that less than 20% of currently known viral protein families are represented in Pfam, a large public collection of profile HMMs from many protein families (see Methods). Furthermore, the viral coverage of Pfam has dropped since new methods for the automated building of profile HMMs were implemented [29]. Additionally, SFams, a recently-released set of profile HMMs used to annotate metagenomic data, do not include any viral sequences [30]. Construction of a comprehensive collection of viral profile HMMs would therefore fill an important gap in the current bioinformatics infrastructure for metagenome annotation. Like similar resources for other domains of life, viral profiles HMMs would also be useful for genome annotation, evolutionary simulations, and studies of individual gene families.

To address this need, we built profile HMMs from the NCBI-curated virally annotated protein sequences in RefSeq [31] and tested the ability of the profile HMMs to correctly classify viral and non-viral sequences as such. We employed a “leave-one-out” cross-validation strategy to assess the degree to which each profile could recall viral sequences that were not used to build the profile, which is the most common situation in viral diagnostics. We found that almost 80% of the HMMs were able to recall 100% of the viral sequences from that gene family before misclassifying any non-viral sequences. Based on these results, we identified a robust subset of HMMs that could recall at least 80% of their constitutive sequences when removed from the profile. Using previously published metagenomic datasets, we compared the performance of profile search using this filtered set of HMMs to pairwise sequence search using BLAST databases. We demonstrated that while BLAST outperforms the profile HMMs for detecting more closely related viral proteins, profile HMMs are more sensitive than BLAST for detecting remote homologs.

## Results

### Building and annotation of viral profile HMMs

**Figure 1:**
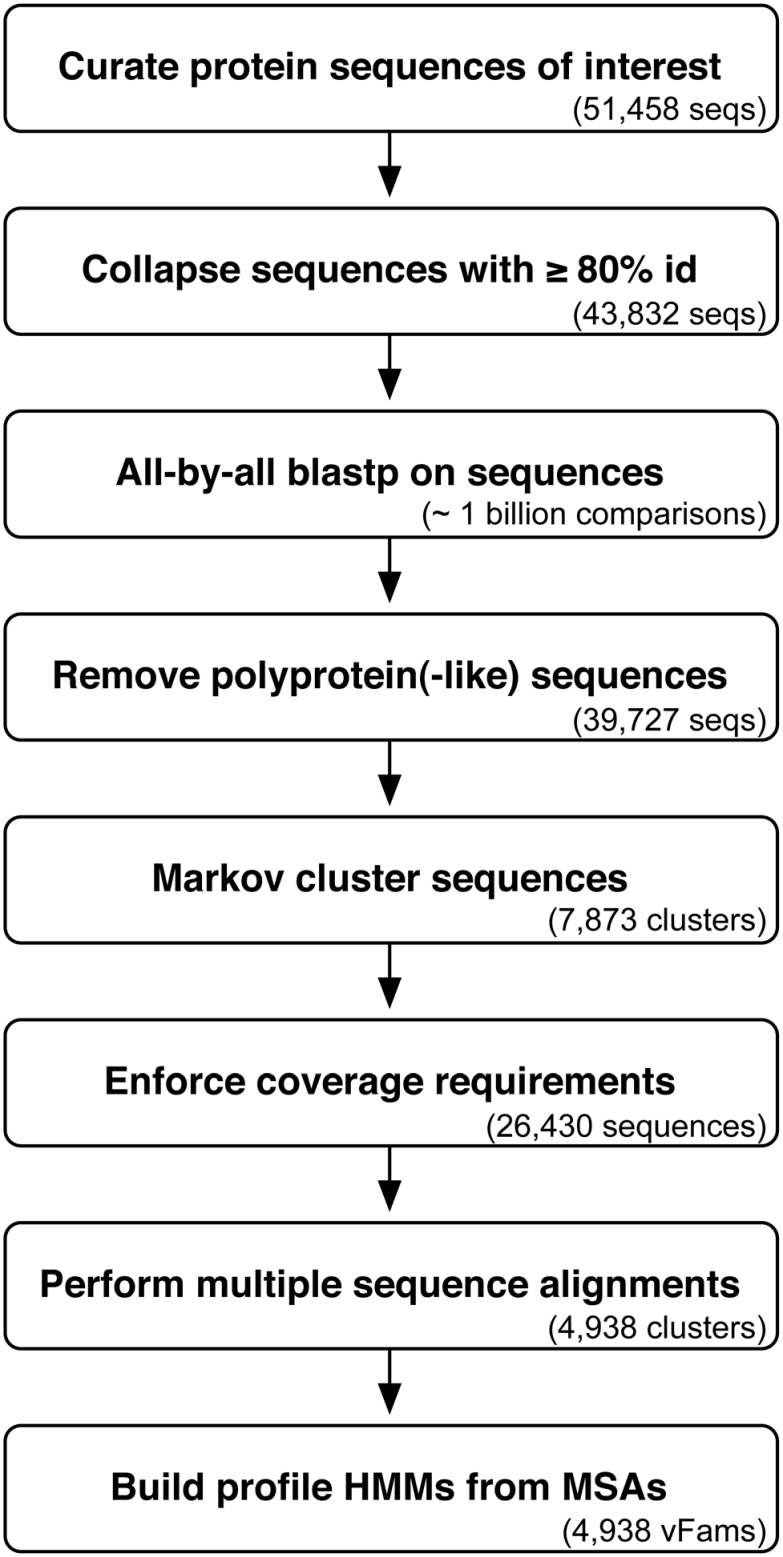
The pipeline for building profile HMMs from a set of curated viral protein sequences. An initial set of protein sequences of interest is curated and reduced by collapsing high-identity sequences. The similarity between all pairs of remaining sequences is calculated using BLAST. Using the BLAST results, polyprotein sequences are inferred and removed. The Markov Clustering algorithm groups the remaining sequences into families. Sequences with extreme lengths are removed before multiple sequence alignments are generated for each family. Multiple sequence alignments are used to train profile HMMs. Statistics for each step in the generation of the vFams are in parentheses.

We developed a bioinformatic pipeline for constructing profile HMMs from all virally annotated (non-phage) proteins in RefSeq [31] (Figure 1; see Methods for details). To ensure the quality of our profile HMMs, we first filtered the 51,458 sequences used as input into the pipeline down to 43,832 sequences by collapsing sequences with 80% or greater identity covering 90% or more of the full sequence. These sequences were further filtered by the removal of polyprotein and polyprotein-like sequences. We used Markov Clustering [32] to group the remaining 39,727 sequences into viral protein clusters, removed single-sequence clusters, and enforced coverage requirements to ensure clustered sequences were close enough in length to one another to produce meaningful multiple sequence alignments [25]. For each of the 4,938 remaining families, we generated a multiple sequence alignment of the clustered proteins and used it to build a profile HMM. These viral protein HMMs were trained from a total of 26,430 sequences that span 72 of 84 viral families (86%), 289 of 321 viral genera (90%), and 1,971 of 2,227 viral species (89%) present in the input sequences retrieved from RefSeq.

To aid downstream annotation and interpretation, each of the profile HMMs (“vFams”) was paired with an annotation file containing basic statistics about the vFam and the sequences used to build it. In addition to profile length, information content, and the number of sequences used to build the profile, the taxonomy of the sequences at the family and genus level was added to aid in attempts at taxonomic classification of reads based on vFam hits. An annotation file is shown in Figure S1.

This standalone viral database of profile HMMs, which we designate “vFam”, likely represents more than five times the number of currently available viral profile HMMs in Pfam; the exact number of viral profile HMMs in Pfam is difficult to assess, as parsing Pfam HMMs by higher levels of taxonomy is neither straightforward nor accurate (see Methods). The vFam database in HMMER3 format, the annotation files, and the software used to build the vFams are freely available at http://derisilab.ucsf.edu/software/vFam and in the public Dryad repository.

### Viral profile HMMs detected unknown viruses in cross-validation experiments

**Figure 2:**
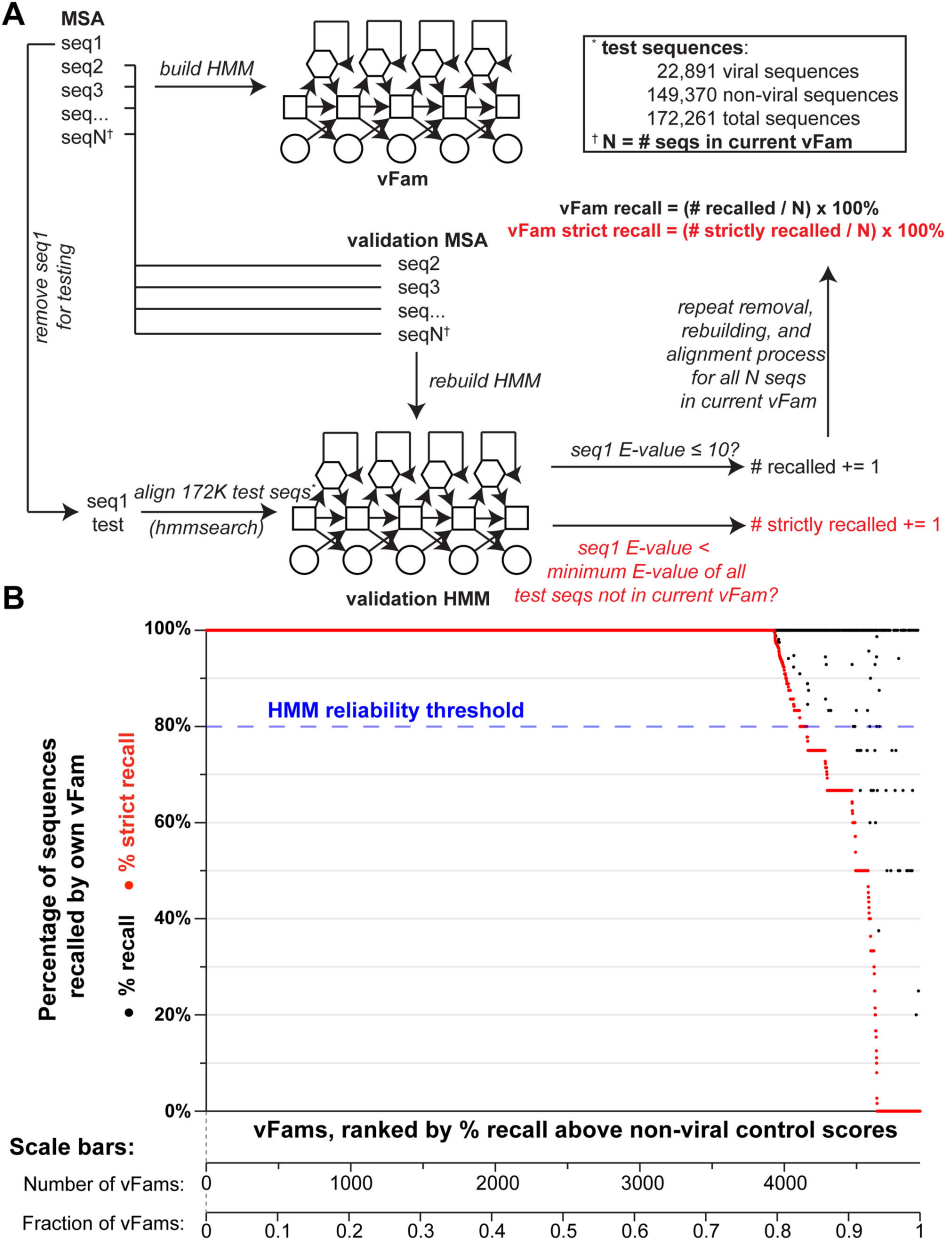
Viral sequence recall for all vFams in cross-validation. (A) A schematic representation of the cross-validation of the vFams is depicted for a single vFam. The initial multiple sequence alignment (MSA) and HMM building are depicted for the vFam being tested (top left). Each sequence is removed from the vFam exactly once, and a validation MSA and validation HMM are built from the remaining sequences. A set of test sequences comprising a large set of non-viral sequences and all viral sequences across all vFams is aligned to the validation HMM, and the left out sequence is evaluated. If the left out sequence is recalled by the validation HMM with an E-value ≤10, the sequence is considered “recalled” by the vFam (black). If the left out sequence is recalled by the validation HMM and additionally has a lower E-value than all test sequences not in the current vFam, the sequence is considered “strictly recalled” (red). The process is repeated for all “N” sequences in the vFam and the vFam’s % recall and % strict recall are calculated. Each vFam was evaluated in this manner. (B) For each vFam in the cross-validation experiments, the percentage of recalled sequences (black) and the percentage of strictly recalled sequences (*i.e.*, E-value less than non-viral controls; red) is plotted. The vFams are ranked by their percentage of strictly recalled sequences (x-axis). A threshold of 80% strict recall (dashed blue line) was used to filter the vFams to the best performing subset. Scale bars below the x-axis show the number and fraction of vFams in the ranked set.

The vFams were designed with the goal of determining whether metagenomic datasets contained any viral sequences, so we employed a “leave-one-out” cross-validation experiment to serve two purposes: 1) to test the robustness of the vFams to ensure they were built from truly homologous proteins and to further determine if they could recruit unknown homologous proteins; and 2) to test each vFam’s ability to accurately distinguish viral from non-viral sequence. Each vFam was evaluated individually. For each vFam, we iteratively removed each sequence from its profile and rebuilt the HMM using the remaining sequences. We used the hmmsearch algorithm in HMMER3 [33] to assess the ability of the vFam to correctly recruit the viral sequences removed from it, simulating the detection of an unknown virus. We additionally compared the E-value of each removed sequence to the E-values for known non-viral sequences as well as the viral sequences from other vFams. These non-viral sequences were drawn from a large collection of ∼150,000 full-length protein sequences from well-studied prokaryotic and eukaryotic organisms, such as *H. sapiens* and *E. coli,* potentially found in the metagenomes of eukaryotic hosts. Each vFam was given a “recall” score, representing the fraction of left-out sequences recalled with an E-value ≤10. Each vFam was additionally given a “strict recall” score, representing the fraction of left-out sequences recalled with an E-value less than all non-viral sequences and viral sequences from other vFams (Figure 2A).

In cross-validation of the full set of vFams, 96% of the sequences were recalled and 91% of the sequences were strictly recalled by the correct vFam. While 4,337 (88%) of the vFams were able to recall 100% of their left-out sequences in cross-validation tests (Figure 2B, black), a subset of 3,931 (80%) of the vFams was additionally able to strictly recall 100% of the left-out sequences (Figure 2B, red). This difference in viral differentiation ability could be attributed to less robust vFams as well as those vFams derived from sequences with non-viral homologs.

vFams comprising viral homologs of host proteins (*e.g.*, enzymes involved in DNA synthesis or post-translational modification, etc.) were anticipated to be uninformative for the classification of sequences as viral or non-viral. Non-robust vFams built from sequences that may not be true homologs of one another could be equally uninformative. We therefore used the results of the cross-validation experiment to filter the set of vFams to those predicted to perform well in the context of experimental metagenomic datasets. Specifically, we retained those vFams that strictly recalled at least 80% of the training sequences in the cross-validation tests (Figure 2B, dashed blue line). We chose a threshold of 80% to maintain broad coverage of viral taxonomy without a large sacrifice in vFam performance. This produced a subset of 4,156 vFams comprising 21,231 sequences, constituting 84% of the number of vFams and 88% of the number of sequences present in the initial set. In an analogous fashion to Pfam-A and Pfam-B, we have dubbed the filtered set and the entire set of HMMs vFam-A and vFam-B respectively [28], [29]. Despite being filtered to a smaller set of vFams and constitutive sequences, vFam-A covers 69 of the 72 (96%) viral families, 283 of the 289 (98%) genera, and 1,930 of the 1,971 (98%) of the viral species present in vFam-B. All downstream analyses were performed with vFam-A.

### vFam recall as a function of profile HMM statistics

**Figure 3:**
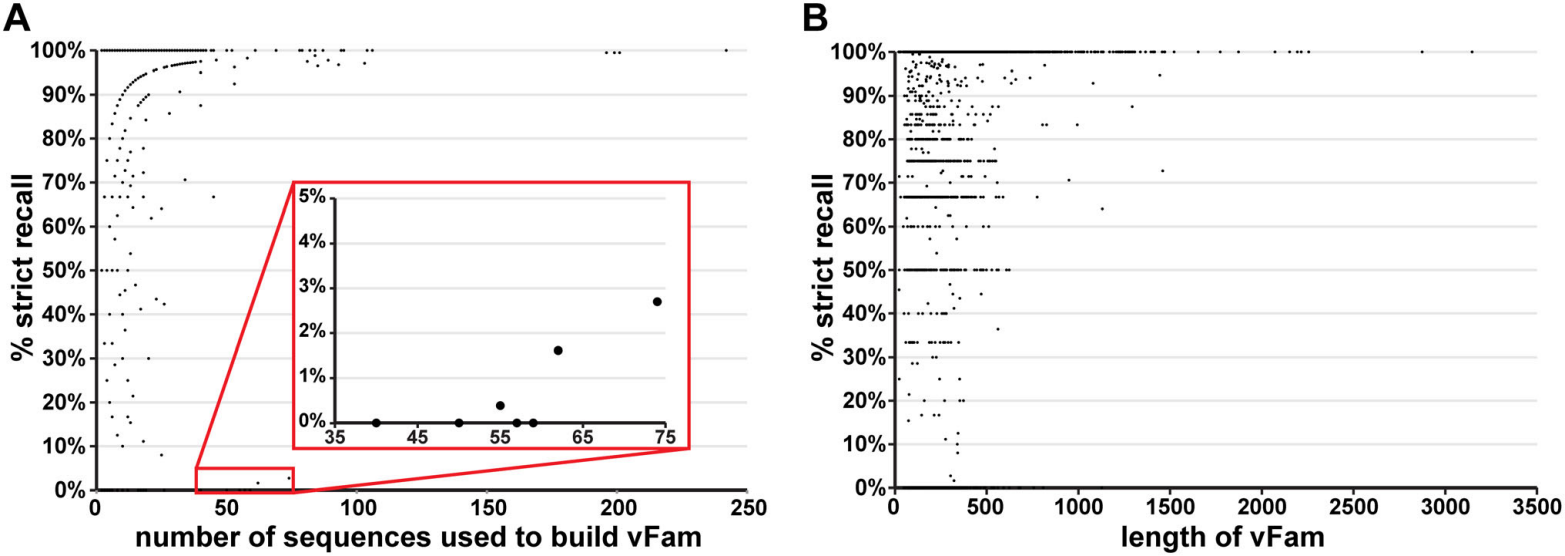
Viral sequence recall as a function of other vFam metrics. For each vFam, the percentage of the vFam’s sequences correctly recalled by the HMM with a score better than all non-viral controls (% strict recall) in the cross-validation experiments is plotted as a function of (A) the number of sequences used to build the vFam; red box (zoomed and inset) highlights HMMs built from 40 or more sequences with strict recall less than 3%, (B) the length of the vFam, (C) the positional relative entropy in the vFam, and (D) the total relative entropy in the vFam.

To further investigate the wide range of recall across the vFams, we sought to identify predictive metrics of vFam performance. To this end, we compared each vFam’s strict recall to the number of sequences used to build the vFam, the vFam’s length, and the presence of viral sequences with non-viral homologs (Figure 3).

Larger vFams (*i.e.,* those built from more sequences) generally displayed higher strict recall than those built from fewer sequences (Figure 3A): while 91% of the vFams built from 10 or more sequences had strict recall of 80% or better, this number dropped to 83% for vFams built from fewer than 10 sequences. All but eight of the 54 vFams generated from 40 or more sequences had strict recall of at least 80% as determined in the cross-validation experiments. Though each of these eight vFams recalled greater than 98% of their left-out sequences, seven of the eight vFams strictly recalled less than 3% of these sequences (Figure 3A, red box). Closer inspection of the sequence clusters used to build these seven poorly performing vFams revealed that they were composed of sequences with non-viral homologs. These seven clusters represented: 1) a set of Inhibitor of Apoptosis (IAP) homologs, proteins conserved in eukaryotes but also present in members of the *Baculoviridae* viral family [34]; 2,3) two clusters containing sequences from two different ribonucleotide reductase subunits (RNR1 and RNR2), enzyme subunits present in a number of different viral families, that are also universally conserved in cellular organisms as an essential enzyme in DNA synthesis [35], [36]; 4) a set of Ankyrin-repeat proteins, which is one of the most common protein domains in higher organisms but is also found repeatedly in genomes of members of the *Mimiviridae* viral family [37]; 5) a set of dUTPases, found in various viral families, but as an enzyme involved in nucleotide metabolism necessary for DNA replication it is also found in many cellular organisms [38]; 6) a set of *Baculoviridae* chitin binding proteins, containing the chitin binding domain found in the matrix proteins of many insects and animals [39]; and 7) a set of Protein-Tyrosine Phosphatases (PTPs), enzymes involved in the post-translational modification of proteins, particularly in signaling transduction pathways of cellular organisms, but that also occur ubiquitously in members of the *Polydnaviridae* viral family that infects insects [40]. All of these vFams were removed during the filtering step as their low strict recall and lack of specificity for viral sequences make them uninformative and potentially misleading when searching for viral sequences in metagenomic datasets. The vFams built from viral sequences with non-viral homologs are present in vFam-B but are not included in vFam-A.

In addition to vFam size and non-viral sequence homology, we examined vFam length as a potential predictor of vFam performance. When comparing strict recall to the length of the vFam, while the longest vFams (or vFams with the most “match states”, which roughly correspond to columns in the multiple sequence alignment) did tend to have high strict recall, there appeared to be almost no correlation between vFam length and strict recall for vFams of length less than 600 (Figure 3B). For vFams of length 600 and greater, 96% had strict recall at least 80%, the filtering threshold employed after cross-validation; for vFams with length less than 600, this number dropped to 83%.

Overall, the major contributing factors to higher vFam strict recall were the number of sequences used to build the vFam and the lack of non-viral homologs of the viral sequences used to build the vFam.

### vFams vs. BLAST on real metagenomic datasets

Many recent viral discovery projects supported by deep sequencing data relied solely on BLAST to identify and classify viral reads [10]–[15]. In order to compare the performance of the vFams to BLAST on real data, we tested both approaches on three previously published datasets containing viruses in metagenomic backgrounds. These three datasets contain viruses that were novel discoveries spanning a range of divergence from previously known viruses, allowing us to explore the sensitivity and precision of vFams and BLAST in different contexts.

#### Human klassevirus 1

**Figure 4:**
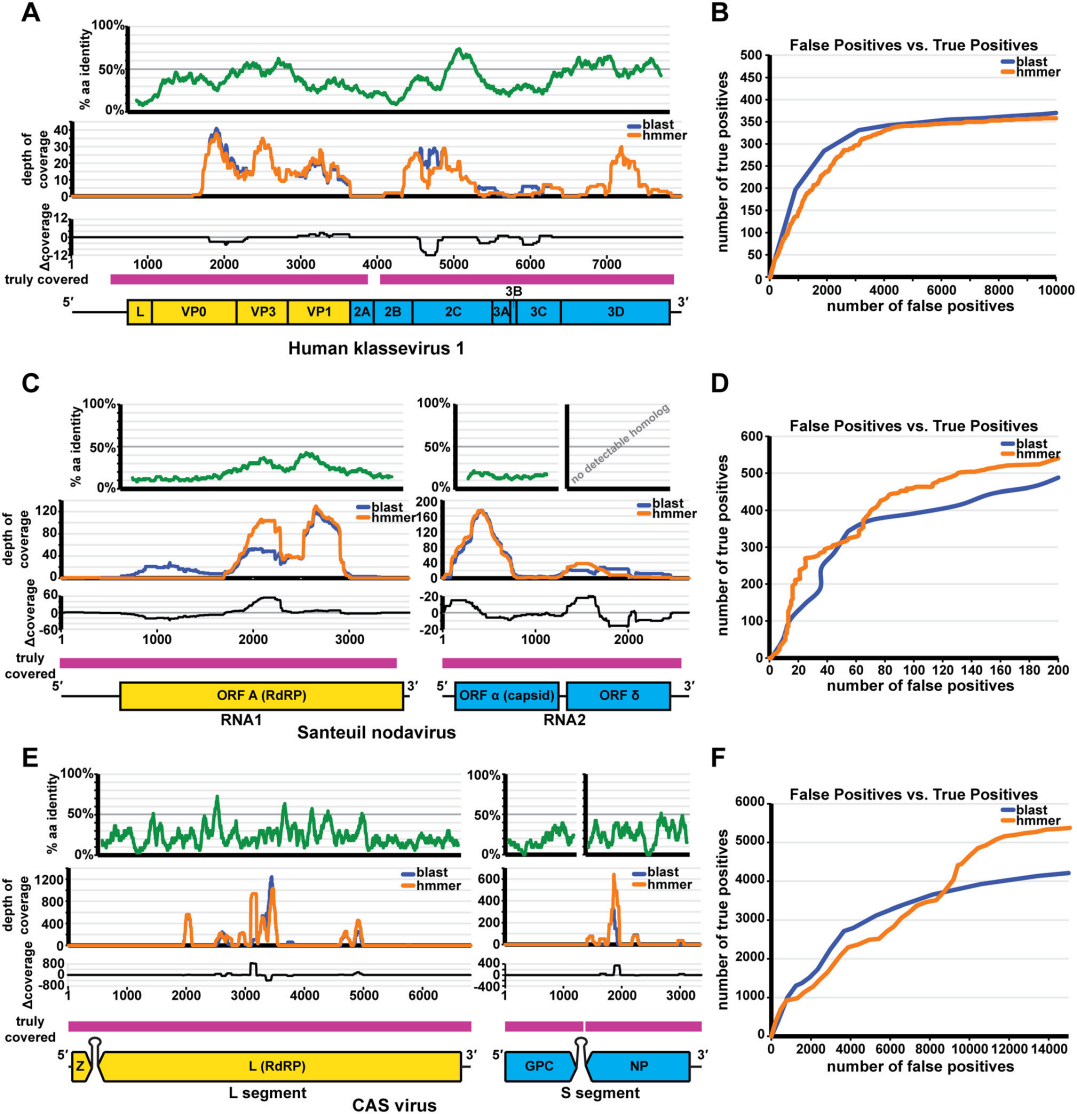
Performance of vFams and BLAST on metagenomic datasets. A comparison of BLAST vs. HMMER for the detection of *Human klassevirus 1*, *Santeuil nodavirus*, and *CAS virus*. (A) Percent amino acid identity for 80 aa windows is shown between the *Human klassevirus 1* and *Aichi virus* polyprotein sequences (green); genome coverage of correctly classified viral reads by BLAST and HMMER is shown in blue and orange respectively; the difference in coverage (HMMER coverage−BLAST coverage = Δ coverage) is shown in black; the regions of the genome truly covered in the full dataset are shown in pink; a to-scale genome schematic of *Human klassevirus 1* is found below, depicting structural proteins (yellow) and non-structural proteins (blue). (B) The number of true positives vs. the number of false positives for the detection of *Human klassevirus 1* is depicted for BLAST (blue) and HMMER (orange). (C) Percent amino acid identity for 84 aa windows is shown between ORF A and ORF α of *Santeuil nodavirus* and the RdRP and capsid proteins of the *Striped Jack Nervous Necrosis virus* (green) [no homolog of ORF δ was detected at the time of the discovery] [42]; genome coverage of correctly classified viral reads by BLAST and HMMER is shown in blue and orange respectively; the difference in coverage (HMMER coverage−BLAST coverage = Δ coverage) is shown in black; the regions of the genome truly covered in the full dataset are shown in pink; a to-scale genome schematic of *Santeuil nodavirus* RNA-1 and RNA-2 is found below, depicting ORF A (yellow), and ORF α and ORF δ (blue). (D) The number of true positives vs. the number of false positives for the detection of *Santeuil nodavirus* is depicted for BLAST (blue) and HMMER (orange). (E) Percent amino acid identity for 33 aa windows is shown between the L protein, the glycoprotein, and the nucleoprotein of CAS virus and the L protein of *Lymphocytic choriomeningitis virus*, the glycoprotein of *Lloviu virus*, and the nucleoprotein of *Lymphocytic choriomeningitis virus* (green) respectively [no homolog of the Z protein was detected at the time of the discovery] [12]; genome coverage of correctly classified viral reads by BLAST and HMMER is shown in blue and orange respectively; the difference in coverage (HMMER coverage−BLAST coverage = Δ coverage) is shown in black; the regions of the genome truly covered in the full dataset are shown in pink; a genome schematic of the CAS virus L segment and S segment is found below, depicting the Z and L proteins (yellow) and the glycoprotein (GPC) and nucleoprotein (NP) (blue) respectively. (F) The number of true positives vs. the number of false positives for the detection of *CAS virus* is depicted for BLAST (blue) and HMMER (orange).

To evaluate the ability of the vFams to detect a novel but less divergent virus, we first analyzed a pool of metagenomes represented by approximately 500,000 reads of 240 nt average length from 141 pediatric cases of diarrhea of previously unknown etiology [11]. There were several known diarrhea-causing viruses identified in the pool, which were removed for the sake of this analysis, as well as 483 reads deriving from a novel 8.0 kilobase picornavirus called *Human klassevirus 1* [11]. The closest known relative of klassevirus by amino acid (aa) identity at the time of its discovery was *Aichi virus* [41] with ∼40% identity across the length of the polyprotein that spans almost the entire genome (Figure 4A). Approximately 7 million read translations were aligned to the viral BLAST database and vFam database, and ranked by BLAST E-value and HMMER3’s domain E-value scores respectively. By comparing the number of true positives against the number of false positives at various scores or better for each alignment method, BLAST and HMMER performed similarly at finding the highest-scoring sequences. BLAST however outperformed HMMER by 5%–20% in terms of sensitivity, or the fraction of all true positives found at a given number of false positives (Figure 4B). This was not surprising because some regions of the klassevirus polyprotein approach 70% pairwise aa identity to proteins in the *Kobuvirus* genus. However, the klassevirus sequences recovered by the vFams covered slightly more of the genome (5,502 nt) than the klassevirus sequences recovered by BLAST (5,472 nt; Figure 4A). While the vFams barely outperformed BLAST in some of the lower percent identity regions of the genome (*e.g.,* VP1), both the vFams and BLAST detected reads from the 2B gene, which represented the most divergent stretch of the genome. Furthermore, BLAST decidedly outperformed the vFams in other lower percent identity regions of the genome (*e.g.*, the 5′ ends of 2C and 3C). While viral genome coverage generally depends both on the ability to detect the virus as well as the initial abundance and subsequent amplification of different genomic regions, this particular example hinted at the ability of the vFams to detect divergent genomic regions at the expense of assigning relatively higher scores to higher identity stretches.

#### Santeuil nodavirus

To evaluate the ability of the vFams to detect a more divergent viral genome, we analyzed a metagenome of 253 nt (average length) reads derived from *C. briggsae* isolated from a snail on a rotting grape in Santeuil, France [42]. Almost 2,500 of the ∼21,000 reads derived from a novel member of the *Nodaviridae* viral family, *Santeuil nodavirus* [42]. The virus comprises two RNA segments, RNA1 (3,628 nt) and RNA2 (2,653 nt). RNA1 contains a single ORF encoding the RdRP which shares ∼26–27% aa identity with related members of the family. RNA2 contains two ORFs, ORF α and ORF δ; ORF α, which encodes the capsid protein, shares ∼30% aa identity with related members of the family, while no homolog was detected for the protein encoded by ORF δ [42]. The percent aa identity between *Santeuil nodavirus* and a related nodavirus, *Striped Jack Nervous Necrosis virus* [43], is depicted in Figure 4C. Almost 400,000 read translations were aligned to both viral databases. While the BLAST approach was able to identify the first 360 high-scoring true positives with virtually identical precision (*i.e.*, the fraction of alignments corresponding to true positives) to HMMER, the rate of true positive identification leveled off and HMMER was 10–15% more sensitive than BLAST at the same number of false positives (Figure 4D) thereafter. BLAST and HMMER were both able to detect the most conserved regions of the RdRP as well as the majority of the capsid sequence (Figure 4C). However, the majority of *Santeuil nodavirus* alignments reported by BLAST constituted “type 0 errors”: 392 of the 777 reads (50.4%) detected by the viral BLAST database that truly derived from *Santeuil nodavirus* aligned to members of viral families other than *Nodaviridae* present in the BLAST database. This accounted for the perceived coverage of ORF δ when no known homolog existed in either database; if BLAST and HMMER were perfectly precise, ORF δ would have no coverage at all. The vFams also produced type 0 errors, though to a lesser extent: 256 of the 732 *Santeuil nodavirus* reads (35%) detected by the vFams aligned to members of viral families other than *Nodaviridae*. The percentage of RNA1 covered by the BLAST true positives (85%) was much higher than the percentage covered by the HMMER true positives (47%), but 100% of the 5′-most BLAST coverage derived from non-specific hits to the viral database. RNA2 was covered to the exact same extent (92%) by both approaches.

#### CAS virus

To evaluate the ability of the vFams to detect a highly divergent viral genome, we analyzed a pool of sequence libraries sampled from six sites (lung, liver, kidney, brain, gastrointestinal tract, and heart) of an Annulated tree boa, *Corallus annulatus*. Approximately 123,000 of the more than 19 million 100 nt reads in the datasets derived from a novel member of the *Arenaviridae* viral family, *CAS virus* (California Academy of Sciences virus). *CAS virus* encodes 4 proteins on two RNA segments [12]. The L segment (6,812 nt) encodes the Z protein and the L protein (RdRP). The S segment (3,368 nt) encodes the glycoprotein-precursor protein (GPC) and the nucleoprotein (NP). Though the polymerase and nucleoprotein have a clear but distant evolutionary relationship to their arenavirus homologs, the glycoprotein precursor’s closest relative is unclear, and there was no detectable homolog for the Z protein (Figure 4E) [12]. Almost 140 million read translations were aligned to both viral databases. While the first ∼950 true positives were found with virtually identical precision by both BLAST and HMMER, BLAST found the next ∼2800 true positives with higher precision than HMMER. However, the rate of identification of true positives vs. false positives started to level off for BLAST while it continued to increase at a relatively constant ratio for HMMER. In fact, the sensitivity for HMMER was ∼25% higher than BLAST, allowing for 15,000 false positives. At this number of false positives, the vFams were not only more sensitive than BLAST, but the true positive reads yielded slightly broader coverage of the genome (though they did cover highly overlapping regions of the genome). The vFam-identified reads covered 24% of the L segment while BLAST-identified reads covered 23%; and for the S segment, the vFam-versus BLAST-identified reads covered 21% and 18%, respectively (Figure 4F).

### Precision of vFams in real metagenomic data analyses

The vFams showed a wide range of precision for the three metagenomic datasets (Table 1). The precision was 3.47% for the detection of *Human klassevirus 1*, 72.95% for *Santeuil nodavirus*, and 26.42% for *CAS virus*. While the true positive rate (TPR) of the vFams was largely a function of sequence divergence from the closest known relatives at the time of discovery, with a higher TPR for the less divergent klassevirus genome and much lower TPRs for the more divergent nodavirus and arenavirus genomes, the false positive rate (FPR) was consistently low across the datasets, never exceeding 2%. The range in precision was largely attributed to the percentage of true positives in each of the datasets: *Human klassevirus 1* was present at 0.09%, *Santeuil nodavirus* was present at 11.99%, and *CAS virus* was present at 0.64% of the initial datasets. By analyzing the TPR and FPR instead of the absolute counts, the vFams indeed performed comparably for each dataset: the ratio of TPR to FPR for each dataset fell in a much narrower range than the precision.

**Table 1:** Statistics on vFam performance on metagenomic datasets.

Virus Dataset	Total Reads	Positive Reads (%)	TP	FP	Precision	FPR	TPR	TPR/FPR
*Human klassevirus 1*	533,453	483 (0.09%)	358	9,957	3.47%	1.87%	74.12%	39.7
*Santeuil nodavirus*	20,787	2,493 (11.99%)	542	201	72.95%	1.10%	21.74%	19.8
*CAS virus*	19,196,993	122,911 (0.64%)	5,369	14,952	26.42%	0.08%	4.37%	55.7

Results for viral detection of *Human klassevirus 1*, *Santeuil nodavirus*, and *CAS virus*. TP = True Positives, FP = False Positives, Precision = TP/(TP+FP), TPR = TP/Positive Reads, FPR = FP/(Total Reads–Positive Reads); TP and FP determined from maximal values in Figures 4B, 4D, and 4F.

Despite the effort to minimize false positives by removing poorly performing vFams, the absolute number of false positives for each dataset was affected greatly by the metagenomic context in which the viruses were identified. By aligning the false positive reads to NCBI’s non-redundant nucleotide sequence database (NT) by nucleotide BLAST and keeping the best scoring alignment with an E-value of 10 or better, we attempted to identify the host of origin for the false positives in each dataset. For the *Human klassevirus 1* dataset, which was derived from a pool of 141 diarrhea samples, over 40% (4377/9957) of the false positive reads had no match to any annotated organisms in NT. The vast majority of the aligned sequences comprised imperfect matches to a variety of bacterial families. For the *Santeuil nodavirus* dataset, which was derived from nematodes and had much higher precision, more than 75% (151/201) of the false positives aligned to a nematode genome; many of these alignments overlapped non-coding regions, resulting in nonsensical translations. For the *CAS virus* dataset, derived from a pool of harvested Annulated tree boa organs, only ∼30% (4584/14952) of the false positives aligned to an annotated organism in NT. Of the sequences that did align, nearly 90% (∼4000) aligned to a snake or other higher eukaryotic organism’s genome in NT.

While identifying true positives in the context of a potentially high number of false positives may be daunting, the signal becomes more apparent by aggregating hits across the vFams (Figure S2). By looking at the taxonomy of the species present in the most frequently hit vFams for each dataset, the cumulative number of alignments to the correct taxa exceeds or rivals the number of false positive alignments to other taxa. Of particular note is the most frequently hit vFam in the *CAS virus* sample, which contains reverse transcriptase homologs; many of the reads aligning to this vFam likely derive from endogenous retroviral elements in the snake genome.

## Discussion

In this work, we constructed vFam, a standalone database of profile HMMs derived from viral proteins, and demonstrated its utility for detecting divergent viral sequences within metagenomic sequence data. Cross-validation experiments on full-length sequences showed high recall for many of the vFams, which covered the vast majority of the known viral taxonomy. When we compared the vFams to BLAST in real metagenomic datasets, the vFams demonstrated an improved detection accuracy when viruses in the dataset were more divergent or when the metagenomic reads acquired through massively parallel sequencing were derived from less conserved regions of the viral genome. Though BLAST exhibited superior accuracy for the detection of high sequence identity matches, we hypothesize that some fraction of datasets currently classified as “virus negative” may in fact contain viruses that were simply too divergent to be detected by BLAST.

In general, we found that the HMM-based strategy had improved precision over the BLAST-based strategy when analyzing metagenomes. However, both methods tended to require tolerance of a high false positive rate to detect true positives. This was primarily due to the difficulty associated with differentiating viral and non-viral sequences. Our statistical curation of vFams to produce vFam-A reduced but did not eliminate this problem: we required that a vFam recalled 80% or more of the cross-validation test sequences with a score better than non-viral sequences and unrelated viral sequences. We tolerated a 20% cross-validation error to maintain broad taxonomic coverage at the expense of retaining some vFams that do not perfectly separate viral and non-viral sequences. For example, in the *Human klassevirus 1* dataset, the majority of false positive alignments we identified derived from bacterial genomes that weren’t well represented in NT. In the *Santeuil nodavirus* and *CAS virus* datasets, non-coding or divergent endogenous retroviral elements from the host genomes led to false positive alignments to the vFams. An additional factor impacting precision was the sequence length of metagenomic data; the probability of spurious alignment decreases as the sequence length increases. Unlike the cross-validation tests that were performed on full-length viral sequences, the metagenomic datasets contained read lengths ranging from 100 to 250 nt, which corresponded to translated ORFs less than 100 aa in length. As the read lengths generated by massively parallel sequencers increase and as sequence assembly algorithms continue to improve, we expect the vFams to perform with increased precision. And as the read lengths further approach and ultimately eclipse the length of typical viral proteins, we expect the precision of the vFams on real metagenomic data to be on the order of what we observed in the cross-validation tests.

Our analysis of real metagenomic datasets supported the conventional wisdom that BLAST is better at finding higher identity matches and can thus aid in more accurate taxonomic assignment, while profile HMMs are better at finding more divergent matches. We propose that the full complement of viral (and non-viral) sequences in metagenomic datasets may be identified using a combination of BLAST and vFams. A straightforward implementation leveraging both search methods could entail 1) a nucleotide BLAST search to a curated set of known non-viral genome sequences (including the host genome, if available) likely to appear in the metagenomic sequence data; 2) a BLAST search to a viral database to capture and taxonomically assign higher identity matches; and 3) a search of the vFams, extending the search space into more divergent territory. To this end, on our download page we provide a FASTA file containing viral protein sequences that can be used as a BLAST database, alongside vFam-A and vFam-B. This approach still presents challenges, however; for example, the noncoding regions of viruses cannot be detected by protein database searching, as translation of sequence from noncoding regions is nonsensical. A more difficult challenge to overcome is the detection of completely novel viral genomes, because both BLAST- and HMM-based methods fundamentally rely on some level of sequence similarity to known viruses. Though the rate of discovery of completely novel viral families is decreasing, the projected number of viral families is expected to continue increasing for at least the next decade [44]. This will demand maintaining up-to-date databases and will place a premium on methods for identifying novel viruses that don’t strictly rely on homology to known viruses. These bioinformatic methods will depend heavily on *de novo* metagenomic assemblers [45] and *ab initio* structural prediction algorithms.

One important downstream application of viral sequences identified using vFams is to aid viral genome assembly from metagenomic data. Nucleating metagenomic assemblies using reads of interest (or reads of unknown origin) in order to produce longer sequences can make the researcher’s job of determining whether a sequence is truly viral or a false positive much easier, quicker, and more inexpensive than the alternative of testing false positives at the bench or critically analyzing each read that aligns to a viral database. Beyond the improvements in sequencing technology that lie ahead, advances in bioinformatic methods will ultimately determine our ability to detect both novel and divergent viruses in the most difficult of cases. In this study, we observed higher recall for those vFams of greater length as well as those built from more sequences. While researchers have no control over the length of viral proteins found in nature, increasing the number and diversity of sequenced viruses will aid the detection of more viruses in the future. Thus, the vFam approach for classifying viral and non-viral sequences will only improve as more viruses covering a greater breadth of the phylogeny are discovered. The combination of pairwise alignment methods with profile HMMs and novel *de novo* sequence assembly methods will provide researchers with a natural workflow to allow progressively more sensitive virus searching of metagenomic sequence data.

## Methods

### Building profile HMMs from viral protein sequences

Protein sequences annotated as viral sequences (taxonomy ID: 10239) were filtered to only those sequences in RefSeq that did not contain the keyword “phage”, and were downloaded from NCBI’s Protein website (http://www.ncbi.nlm.nih.gov/protein) in FASTA [46] format in February 2013. These 51,458 sequences were used as input to a software pipeline written in Python (Figure 1). Sequences with greater than 80% pairwise identity and greater than 90% mutual coverage were collapsed as is standard for Pfam profile HMMs [28], [29], using CD-HIT [47]. Remaining sequences were aligned “all-by-all” using protein BLAST (blastp) [16]. To allow proteins derived from polyprotein sequences to be represented in profiles with their homologs, and not with all protein products from all related polyproteins, polyprotein and polyprotein-like sequences were identified and filtered out of the sequence set. Sequences longer than 400 amino acids in length were identified as polyprotein or polyprotein-like if at least 70% of the sequence length was covered by two or more other proteins in the sequence set that were covered at least 80% by the longer sequence. The remaining sequences were grouped into potential profile groups by Markov Clustering (MCL) [32] using the default inflation number of 2.0. In order to build high-quality multiple sequence alignments, bidirectional coverage requirements were enforced as previously described [25], with a sliding coverage scale from 60% for sequences shorter than 100 amino acids to 85% for sequences longer than 500 amino acids. Multiple sequence alignments (MSAs) were produced in the aligned-FASTA format by MUSCLE [48], and profile HMMs (“vFams”) were built from the MSA aligned-FASTA files using HMMER3’s hmmbuild tool [33].

### Viral classification sequence set

In order to assess and compare the ability of the vFams to distinguish their constitutive viral sequences from non-viral sequences and unrelated viral sequences, we downloaded a test set of ∼150 K sequences from NCBI. This set included RefSeq-curated sequences from well-annotated and commonly-studied prokaryotic and eukaryotic organisms from the following species: *Arabidopsis thaliana, Escherichia coli, Drosophila melanogaster, Homo sapiens, Mus musculus, Streptomyces coelicolor A3(2), Saccharomyces cerevisiae*, and *Staphylococcus aureus*. For the sake of classification, these sequences were considered “negative”, as were those sequences derived from other vFams. As each vFam was being cross-validated, the left-out sequence under consideration was deemed “positive”. All sequence alignments were performed with HMMER3’s hmmsearch tool, using the default alignment parameter values in addition to outputting the tabular domain output format. The union of the non-viral sequences and the viral sequences included in the vFams formed the test set for the cross-validation experiments.

### Leave-one-out cross-validation

To validate the vFams for the detection of pseudo-novel sequences (*i.e.*, not used to build the profiles), we performed a leave-one-out cross-validation experiment. For each profile, each full-length sequence was pulled out of the multiple sequence alignment used to build the vFam, a new validation profile HMM was constructed from the subsequent alignment, and this amended profile HMM’s ability to correctly classify the removed viral sequence versus non-viral and unrelated viral sequences was assessed. For profiles with fewer than 10 sequences, the multiple sequence alignments were rebuilt in the absence of the left-out sequences before building the validation profile HMMs. For profiles with 10 or more sequences, the left-out sequences were simply removed from the previously built multiple sequence alignment files before building the validation profile HMMs, because large alignments should be relatively insensitive to the left-out sequence. Each variant of each profile HMM was searched by all sequences in the test set using hmmsearch. After only keeping the best hit for each query sequence, the recall of each vFam was calculated as the percentage of sequences that was recalled by the profile built without the target sequence. Additionally, a stricter version of the recall of each vFam was calculated as the percentage of sequences that was recalled by the profile built without the target sequence that scored the left-out target sequence better than all non-viral and unrelated viral test sequences (Figure 2A).

### Deep sequencing reads translation

Because HMMER3’s hmmsearch tool only accepts protein sequences as targets for comparison to profile HMMs, we created a tool to translate the deep sequencing nucleotide reads into protein sequences. DNA sequences were translated in 6 frames using TranslatorTI (software implemented for this study; available from http://derisilab.ucsf.edu/software/vFam/) using the standard genetic code. TranslatorTI supports the full range of ambiguous IUPAC nucleotide codes, so codons with ambiguous nucleotides were translated if allowed by the codon redundancy of the genetic code. TranslatorTI splits up translated amino acid (aa) sequences using stop codons (‘*’) and ambiguous amino acids (‘X’) as delimiters to produce translated open reading frames (ORFs) from the input reads. We used all ORFs that encoded for 10 or more residues in subsequent HMMER and BLAST searches.

### Previously published viral metagenomic datasets

To compare profile HMM alignment to pairwise sequence alignment performance by BLAST on real viral metagenomic data, we used three previously published datasets. The first dataset derived from a pool of 141 diarrhea samples of previously unknown etiology in Northern California (http://dx.doi.org/doi:10.7272/q61z429d). A novel member of the *Picornaviridae* viral family, *Human klassevirus 1*, was discovered in the 540,412 unique reads with an average length of 240 nt, generated on the Roche Genome Sequencer FLX platform [11]. The reads were stripped of sequences mapping with 90% or greater identity to known diarrhea-causing viruses: 6,959 reads mapped to the *Adenoviridae* and *Caliciviridae* viral families by translated BLAST (blastx) [16]. Of the remaining 533,453 reads, 483 derived from *Human klassevirus 1*. Translation of the reads produced 7,088,743 protein sequences 10 aa and longer. Sequences from the nearly identical *Salivirus* were removed from the BLAST database to simulate the state of the database before the discovery of *Salivirus* and *Human klassevirus 1*, and vFams containing *Salivirus* sequences were replaced with the profile HMMs built without *Salivirus* sequences that were generated during the leave-one-out cross-validation experiments.

The second dataset derived from *C. briggsae* isolated from a snail on a rotting grape sampled in Santeuil, France (http://dx.doi.org/doi:10.7272/q65q4t1r). A novel member of the *Nodaviridae* viral family, *Santeuil nodavirus*, was discovered in the 20,787 reads with an average length of 253 nt, generated on the Roche Titanium Genome Sequencer [42]. Of the ∼21,000 total reads, 2,493 reads derived from *Santeuil nodavirus*. Translation of the reads produced 397,125 protein sequences 10 aa and longer. Sequences from *Santeuil nodavirus* were removed from the BLAST database to simulate the state of the database before the discovery of the virus, and vFams containing *Santeuil nodavirus* sequences were replaced with the profile HMMs built without *Santeuil nodavirus* sequences that were generated during the leave-one-out cross-validation experiments.

The third dataset derived from a pool of sequence libraries sampled from six sites (heart, gastrointestinal tract, brain, kidney, liver, and lung) of an Annulated tree boa (SRA accessions: SRX170642, SRX170636, SRX170629, SRX170623, SRX170616, SRX170609). Of the 19,196,993 100 nt reads (analysis was only performed on the first read of each read pair), 122,911 were derived from a novel member of the *Arenaviridae* viral family, *CAS virus* (California Academy of Sciences virus) [12]. Translation of these reads produced 139,690,696 protein sequences 10 aa and longer. Sequences from *CAS virus* and the simultaneously discovered *Golden Gate virus* were removed from the BLAST database to simulate the state of the database before the discovery of these viruses. Three vFams contained sequences from both novel arenaviruses: two of the profile HMMs were rebuilt from a multiple sequence alignment generated after removing both viruses from the underlying sequence clusters, and a third profile HMM corresponding to the glycoprotein was removed altogether from the HMM database because *CAS virus* and *Golden Gate virus* glycoproteins were the only two sequences present in the cluster, and their removal rendered the cluster an empty sequence set.

For each dataset, blastp was run with default parameters and E-values were used to score alignments. To ensure HMMER3 alignment of shorter sequences by allowing suboptimal seeding of alignments, the inclusion thresholds for the heuristic throttles in hmmsearch were adjusted (–F1 0.02 –F2 0.02 –F3 0.02). To adjust for bias in the hmmsearch scoring function due to variability in the lengths of the profile HMMs, we used the domain-specific E-value scores (from the –domtblout output) instead of the full sequence E-value scores.

### Alignment and taxonomic classification of false positive reads from viral metagenomic analysis

To taxonomically classify the false positive non-viral reads that aligned to vFams, the reads were aligned to NCBI’s non-redundant nucleotide sequence database (NT; December 2013) using nucleotide BLAST (blastn version 2.2.25+) with default parameters. Only the highest-scoring alignment was kept for each aligned query sequence, and the respective subject sequence’s GenInfo Identifier was used to pull the associated Taxonomy ID (TaxID) from the NCBI Taxonomy databases (ftp://ftp.ncbi.nih.gov/pub/taxonomy/). Each TaxID’s full taxonomic lineage was queried from the NCBI Taxonomy databases, and the taxonomic information for all false positives was aggregated at the division, family, genus, and species level to count the number of reads aligning to each taxon at each level of taxonomy. Alignments of interest were more critically inspected to deduce the likely reason for being falsely classified as viral by the vFams.

### Estimation of the number of Pfam viral profile HMM entries

Due to the difficulty of parsing Pfam profile HMM entries by higher levels of taxonomy, *i.e.*, the Superkingdom “Viruses”, we estimated the number of viral HMMs based on the keyword searches of “virus” and “viral”, which yield 523 and 348 Pfam domains respectively, of which 131 are overlapping and 737 are unique. These 737 profile HMMs may represent an overestimate of the actual number of purely viral HMMs, as Pfam houses domains that contain both viral and non-viral homologs.

## Supporting Information

Figure S1 **Example annotation file for a vFam.** Each vFam has an associated annotation file containing information including the FASTA titles and total number of sequences used to generate the vFam, the length of the vFam, and the total and positional relative entropy of the vFam. Associated taxonomic information is also provided at the family and genus level to aid downstream taxonomic classification.
(EPS)

Figure S2 **Histogram of vFams producing alignments in metagenomic dataset analyses.** A histogram of the top 100 vFams producing alignments and the number of sequences aligned to each of these vFams from the metagenomic dataset analysis for (A) *Human klassevirus 1*, (B) *Santeuil nodavirus*, and (C) *CAS virus*. Red asterisks denote the alignments for vFams containing true homologs of the virus found in the metagenomic dataset.
(EPS)
